# Morpho-Molecular Identification of *Fusarium equiseti* and *Fusarium oxysporum* Associated with Symptomatic Wilting of Potato from Pakistan

**DOI:** 10.3390/jof10100701

**Published:** 2024-10-08

**Authors:** Arsh Bibi, Fathia Mubeen, Ali Rizwan, Irfan Ullah, Masooma Hammad, Muhammad Abu Bakar Waqas, Ayesha Ikram, Zaheer Abbas, Dennis Halterman, Nasir Ahmad Saeed

**Affiliations:** 1 Agricultural Biotechnology Division, National Institute for Biotechnology and Genetic Engineering College, Pakistan Institute of Engineering and Applied Sciences, Faisalabad 38000, Pakistan; arshashuali@gmail.com (A.B.);; 2 Soil and Environmental Biotechnology Division, National Institute for Biotechnology and Genetic Engineering College, Pakistan Institute of Engineering and Applied Sciences, Faisalabad 38000, Pakistan; 3 Department of Biotechnology and Genetic Engineering, Hazara University Mansehra, Mansehra 21300, Pakistan; 4 Industrial Biotechnology Division, National Institute for Biotechnology and Genetic Engineering College, Pakistan Institute of Engineering and Applied Sciences, Faisalabad 38000, Pakistan; 5 National Institute for Genomics & Advanced Biotechnology, National Agriculture Research Center, Park Road, Islamabad 45500, Pakistan; 6 Department of Agriculture–Agricultural Research Service, Madison, WI 53706, USA

**Keywords:** potato wilt, first report from Pakistan, morphology, molecular identification, phylogenetic analysis, biocontrol, pathogenicity

## Abstract

Potato (*Solanum tuberosum* L.) is one of the emerging staple crops in Pakistan, with Punjab producing over 95% of the country’s potatoes. Wilt is an emerging threat to the potato crop worldwide, including in Pakistan. We identified and characterized *Fusarium* species associated with potato wilt in Pakistan through morphological and molecular analyses. Samples were collected during the 2020–2022 potato seasons from five major potato-growing regions: Sahiwal, Chichawatni, Pakpattan, Kamalia, and Faisalabad. Morphological characterization, *internal transcribed spacer* (*ITS*) sequencing, specific *translation elongation factor 1-alpha* (*TEF*) sequencing, and phylogenetic analysis were used to identify six different *Fusarium* species: *F. oxysporum*, *F. equiseti*, *F. incarnatum*, *F. fujikuroi*, *F. annulatum* and *F. thapsinum*. Pathogenicity tests in a greenhouse revealed that *F. oxysporum* and *F. equiseti* were responsible for Fusarium wilt in all sampled regions, with *F. oxysporum* being more prevalent in wilted samples. This is the first report of *F. equiseti* on wilted potatoes in Pakistan. In vitro biocontrol tests using *Trichoderma harzianum* showed 89% inhibition against *F. equiseti* and 65% inhibition against *F. oxysporum*. These findings on *F. equiseti* will aid in developing future control strategies, including biocontrol measures for Fusarium wilt in potatoes.

## 1. Introduction

Potato (*Solanum tuberosum* L.) is the third most economically important staple crop worldwide, after rice and wheat [1]. In Pakistan, three potato crops are produced annually in the spring, summer, and autumn, depending on the temperature of that region. Potatoes are grown in different ecological conditions in Pakistan, from hilly to plain areas [2]. Pakistan is the ninth largest producer of potatoes worldwide, with 7.94 million tons of production, according to official data for 2022–2023 [3]. Potato tubers are a rich source of water, carbohydrates, vitamins, mineral elements, proteins, and fats and play an essential role in the food security of developing countries, which produce more than half of the total world production [4,5]. The crop is popular due to its nutritional quality and easy availability in Pakistan and elsewhere [5,6].

Potato production needs to meet demand in Pakistan and falls behind neighboring Bangladesh and India [7]. Lower production is due to several abiotic and biotic stresses and relatively limited land available for potato cultivation. Biotic constraints drastically impact the growth and production of potatoes and include low-yielding varieties and diseases caused by viral infections, bacterial wilt, oomycete, fungal pathogens, and nematode parasites [8]. These constraints are prevalent in Pakistan and cause seasonal fluctuations in potato production [9].

Fungal pathogens cause considerable losses in potato production from field to storage [10]. The genus *Fusarium* is one of the most essential mycotoxigenic fungal genera of phytopathogenic fungi, which causes wilting of stems and leaves in the field and tuber dry rot during storage [11]. Fusarium wilt is a vascular disease caused by several *Fusarium* species [12,13].

Species of *Fusarium* are filamentous, hyaline fungi most commonly found in soil, water, plants, and organic substrates [13,14]. Taxonomic classification of *Fusarium* species is an ongoing and dynamic process (www.fusarium.org, accessed on 7 September 2024). Approximately two hundred *Fusarium* species have been identified [15]. *Fusarium* spp. infect plants through roots and then colonize xylem vessels of the stem [16]. Fusarium pathogens survive in plant parts and can remain viable in soils for many years. Fusarium wilt symptoms include yellowing of leaves (chlorosis), necrosis primarily on leaf margins that curl slightly, stunting of growth, and wilting of leaves. Stems usually remain a dark, dull green after leaves wilt. Vascular tissue of stems, roots, and leaf petioles turn brown with some brownish flecking of the pith [17,18].

*Fusarium equiseti* is a *Fusarium* species associated with chili wilt [19]. It is reported as a weak pathogen affecting cereals and is rarely found to be related to Fusarium head blight-diseased kernels [20]. This species is commonly found in tropical and sub-tropical areas [21]. However, the pathogenicity of this species has been underestimated. The species belongs to the *F. incarnatum–F. equiseti* complex and is genetically diverse [19]. They are economically important species, as they caused severe vascular wilts and root rot diseases in various crops, including potatoes [22]. The *F. oxysporum* isolates are very diverse and exhibit considerable variation in their cultural, morphological, and pathogenic characteristics. In culture, *F. oxysporum* produces colorless to pale yellow mycelia that turn pink or purple with age. Potato plants infected by *F. oxysporum* exhibit yellowing, wilting, and drying that eventually lead to the death of the leaves. The wilting of plants may be caused by toxins produced by the fungus [23,24,25,26,27,28,29]. The *F. oxysporum* spores germinate to form mycelia, which enter the roots and find their way to xylem vessels [26]. Ultimately, the plant will wilt and die. Infected plant parts remain in the soil and can serve as a source of inoculum for future crops [24]. Disease prevalence of Fusarium wilt (*Fusarium oxysporum* f.sp. *lycopersici*) in tomatoes was determined in the primary tomato-growing regions within Punjab, Pakistan [30]. The 92 isolates of *Fusarium oxysporum* were morphologically identified from chili pepper in Punjab, Pakistan [31].

Integrated disease management practices are traditionally applied to tackle these biological stress factors influencing potato production [32]. They include using disease-free seeds or resistant varieties, environmental monitoring, and chemical pesticide applications [33]. Conventional control measures include certified pathogen-free seeds and long crop rotation [34]. Using resistant varieties is the most appropriate method to control soil-borne diseases [32]. In the absence of resistant varieties, chemical control is an alternative to combat disease. Bio fungicides and biocontrol agents such as *Trichoderma* species can also effectively manage Fusarium wilt [13].

This study aims to address the significant threat posed by Fusarium wilt to potato crops in Pakistan by identifying the specific *Fusarium* species responsible for the disease. By utilizing sequencing of PCR-amplified *internal transcribed spacer* (*ITS*) regions and specific *translation elongation factor 1-alpha* (*TEF*) regions, we seek to accurately determine the *Fusarium* species associated with potato wilt. Additionally, we will evaluate the pathogenicity of the primary culprits, *F. oxysporum* and *F. equiseti*, to understand their impact on potato plants. Furthermore, we will explore the potential of *Trichoderma harzianum* as a biocontrol agent against these pathogens in vitro. This comprehensive approach will contribute to the development of effective control strategies and enhance the management of Fusarium wilt in potato crops in Pakistan.

## 2. Materials and Methods

### 2.1. Sampling, Isolation, and Morphological Characterisation of Fungal Cultures

Symptomatic leaves showing browning and necrotic spots were collected from Kamalia (30°46′00.3″ N 72°36′48.1″ E), Chichawatni, (30°35′45.2″ N 72°48′29.6″ E; 30°46′00.3″ N 72°48′29.6″ E), Sahiwal (30°42′51.3″ N 73°04′48.5″ E; 30°42′51.6″ N 73°04′48.4″ E; 30°45′58.6″ N 73°00′44.5″ E; 30°45′58.9″ N 73°00′44.7″ E), Pakpattan (30°25′14.8″ N 73°31′40.8″ E), and Faisalabad (31°23′36.71″ N 73°2′6.14″ E) in polythene bags from mid-season (February) to the harvesting season (April) in 2021 and 2022. All the samples were brought to the Gene Transformation lab at the National Institute for Biotechnology and Genetic Engineering (NIBGE) and stored at 4 °C before processing. The infected potato leaves were washed with tap water to remove soil particles. Infected leaves were then surface sterilized with 1% mercuric chloride (Millipore Sigma, Burlington, VT, USA) for 3 min, followed by three consecutive washes in distilled water. Leaves were cut into small pieces containing the necrotic lesion and living tissue. Four leaf pieces were placed on Petri plates containing potato dextrose agar (PDA) (Merck KGaA, Darmstadt, Germany) amended with ampicillin (20 mg/mL) and rifampicin (75 mg/mL; Thermo Fisher Scientific, Waltham, MA, USA). All the plates were sealed with parafilm tape (Pechiney, Paris, France), labeled with an isolate code and date of isolation, and incubated at 25 °C with 12 h of light per day for 4–7 days.

### 2.2. Morphological Identification

Different fungal colonies were observed on the PDA medium, but only typical colonies with *Fusarium* features were selected. Each isolate was purified using a hyphal tip and single-spore technique [35]. Morphological characteristics (e.g., spore shape, mycelium septation, colony form, colony color, colony diameter) of isolates were examined using a binocular compound microscope (Bausch & Lomb, Laval, Canada). The diameter of the colony was measured in mm after seven days (Table 1) The morphological features were confirmed by comparing them with the published literature [22,36].

### 2.3. DNA Extraction for Molecular Characterization

After 7–10 days of incubation, mycelia from the media were scraped with a sterile scalpel and ground into a fine powder in liquid nitrogen using a mortar and pestle. The genomic DNA of *Fusarium* isolates was extracted using the cetyl trimethyl ammonium bromide (CTAB) method [37]. The crushed sample was placed into a 1.5 mL centrifuge tube containing 700 µL of CTAB buffer. Samples were incubated in a water bath (Polysciences, Warrington, PA, USA) at 65 °C while mixing every 10 min. After 30 min, samples were cooled to room temperature, and 700 µL of 24:1 chloroform/isoamyl alcohol (Research Organics, Cleveland, OH, USA) and 2.5 µL of RNAse (Thermo Fisher Scientific, Waltham, MA, USA) were added. Samples were mixed by inverting and shaking, followed by centrifugation (Hermle Benchmark, Franklin, TN, USA) for 15 min at 13,000 rpm. The upper layer was transferred into a new autoclaved 1.5 mL centrifuge tube containing 500 µL of isopropanol (Millipore Sigma, Burlington, VT, USA) and 1/10 vol 3 M sodium acetate (Thermo Fisher Scientific, Waltham, MA, USA). The solution was mixed and stored at −20 °C for 30 min. Samples were centrifuged for 15 min at 13,000 rpm to pellet the DNA. The supernatant was discarded, and the pellet was washed with 200 µL of 70% ethanol (Millipore Sigma, Burlington, VT, USA), followed by centrifugation for 2 min at 13,000 rpm. DNA pellets were dried at 37 °C for 15 min and dissolved in 70 µL of sterilized distilled water.

### 2.4. PCR Amplification and Sequencing

PCR amplification of the *internal transcribed spacer* (*ITS*) region was carried out using primer set ITS1, 5′-TCCGTAGGTGAACCTGCGG-3′, and ITS-4, 5′-TCCTCCCGCTTATTG ATATGC-3′ [38], and for *translation elongation factor 1-alpha* (*TEF*) using primer set TEF F 5′-ATGGGTAAGGAGGACAAGAC-3′ and TEF R 5′ ACGGGTTAGCATGAGAAGGT-3′ [39,40]. A 25 µL mixture was used for amplification, and each mixture contained 15 µL of Dream Taq DNA polymerase (Thermo Fisher Scientific, USA), 9 µL of sterile distilled water, 2 µL of 50 ng/µL genomic DNA, and 1 µL each of 10 µM reverse and forward primers. The PCR mixture was incubated in a thermal cycler (Bio-Rad, Hercules, CA, USA) and programmed with an initial denaturation at 95 °C for 5 min, followed by 35 cycles of amplification consisting of denaturation at 95 °C for 30 s, an annealing temp of 55 °C for 30 s and extension at 72 °C for 45 s, followed by a final extension at 72 °C for 10 min. The amplified product (2 µL) was loaded on 1% agarose gel with ethidium bromide and observed using a UV gel documentation system (Bio-Rad, Hercules, CA, USA). The amplified PCR products were cleaned using a Monarch PCR DNA Cleanup kit (New England Biolabs, Ipswich, MA, USA) and cloned into PTZ using a Fermantas Insta TA clone PCR cloning kit according to the manufacturer’s instructions. The presence of the inserts was confirmed by *Sma1* and *Xba1* (Thermo Fisher Scientific, Waltham, MA, USA) restriction enzyme digestion and by PCR amplification using M13 F 5′-GTAAAACGACGGCCAGT and M13 R 5′-CAGGAAACAGCTATGAC primers. The purified PCR product was sequenced using the Sanger sequencing method (Functional Biosciences, Madison, WI, USA). The same primers used for PCR amplification were also used for sequencing.

### 2.5. Sequence Analysis and Alignment

The sequences were assembled using BioEdit software version 7.7.1 to produce complete contigs from forward and reverse sequences. Basic local alignment search tool (BLAST v. 11.0.13) analysis was performed to check the sequence similarities of sequenced isolates with already-deposited sequences (reference sequences) in the National Center for Biotechnology Information (NCBI). The species names were assigned to the isolates after comparison with the representative sequences available in NCBI. The isolate sequences were submitted to GenBank, and accession numbers are listed in Table 2. The sequences were aligned using Clustal W in MEGA11 version 11-0-13 [41].

### 2.6. Phylogenetic Analysis

To resolve the relationship among *Fusarium* isolates, a phylogenetic tree was created by using the Maximum Likelihood method and Jukes–Cantor model for *ITS*, *TEF-1α* [41,42]. The percentage of trees in which the associated taxa clustered together in the bootstrap test (1000 replicates) is shown next to the branches. Initial tree(s) for the heuristic search were obtained automatically by applying Neighbor-Join and BioNJ algorithms to a matrix of pairwise distances estimated using the Jukes–Cantor model, and then selecting the topology with superior log likelihood value. All positions containing gaps and missing data were eliminated (complete deletion option). Evolutionary analyses were conducted in MEGA11 [41]. Reference sequences used in analysis are according to [43].

### 2.7. Pathogenicity Assay to Confirm the Virulence of F. oxysporum and F. equiseti

*Fusarium oxysporum* and *F. equiseti* inocula were grown in potato dextrose broth (Sigma-Aldrich). After five days of incubation at 25 °C in a shaker incubator, the purity of the culture was checked by microscopic visualization. The culture was filtered through cheese cloth and centrifuged for 2 min at 12,000 rpm at room temperature. The pellet was dissolved in 10 mL of autoclaved water, and spores were counted using a hemocytometer. A total volume of 5 mL of inoculum was applied to the potato variety Kuroda. Fungal inoculum was applied following Ford (2003) with a modification that involved using 220-grit silicon carbide paper (3M Science. Applied to Life, Maplewood, NJ, USA). In total, 5 ml of inoculum was applied to silicon carbide paper and gently pressed against the abaxial surface of leaflets near the middle of the plant. Three leaflets per plant were inoculated for this purpose. Plants were kept in a glass house at 25–27 °C and 16/8 h light/dark in pots with three replicates. Plants were visually analyzed and rated as resistant, moderately resistant, susceptible, and severely susceptible by observing the symptom severity visually seven days following inoculation. An ordinal rating scale of 0–4 was used, where 0 = healthy plant leaves (resistant), 1 = yellowing of plant leaves (moderately resistant), 3 = browning/wilting of plants (susceptible), and 4 = blackening/death of plants (severely susceptible). Symptomatic leaves were collected for re-isolation, and isolates were confirmed by sequencing the *ITS* region as described previously. A pathogenicity test for wilt was conducted for all the isolates of the study in replication. Symptoms of yellowing and wilting of leaves on each stem were noted for all the inoculated replicates and control plants. The disease index was calculated using the formula:

$$Disease\;Index\;(DI)=\frac{\sum \;(Severity\;score\;of\;each\;plant)}{maximum\;severity\;score\;X\;number\;of\;plants}\;X\;100$$

where severity of symptoms was rated by the following criteria: no symptoms, healthy plants = 0; yellowing on 25% of plant = 1; yellowing and blackening on 25–50% of plant = 2; yellowing and blackening on 50–75% of plant = 3; blackening on 75–100% of plant (plant death) = 4

### 2.8. Effect of F. equiseti and F. oxysporum on Tuber Production

*F. equiseti* and *F. oxysporum* were used to inoculate Kuroda, AGB red, AGB pink, and AGB CH2 to determine the effect on tuber production. Plants were grown in a greenhouse with a controlled temperature of 25–27 °C and a 16/8 h light/dark period in pots with three replicates. Plants were inoculated with pathogens using silicon carbide paper as described previously. Plants were observed visually as the symptoms appeared. Tubers of each potato genotype were also grown in replicates in a field in October 2023 at NIBGE, Faisalabad. Plants were grown in 6 rows per plot with 6 plants in each row. Two rows were used as a non-inoculated control for AGB red and Kuroda. Two rows were used to inoculate AGB red and Kuroda with *F. oxysporum*, and two rows were used to inoculate the two host genotypes with *F. equiseti*. Plants were inoculated using an abrasive sheet as described previously. Plants were inoculated after 4 weeks of cultivation. Potato tubers were harvested in February 2024. Plant tuber data were collected, including the number of potatoes per plant and their average mass.

### 2.9. In Vitro Antagonistic Activity of Trichoderma Harzianum

For the in vitro evaluation of the antagonistic potential of *Trichoderma harzanium,* strains named T.A, T.B, and T.C, obtained from the Gene Transformation lab (NIBGE), were tested against *F. oxysporum* and *F. equiseti* using a dual-culture technique [44]. Fungal pathogens were separately grown on PDA for 5–7 days at 25 ± 2 °C. Likewise, *T. harzianum* was grown on PDA plates at 25 ± 2 °C for five days. Mycelial disks (8 mm diameter) of pathogens and antagonistic strains were inoculated onto fresh PDA plates, such that the pathogen was at one edge and the antagonist on the other side of the plate, both at a 1.5 cm distance from their respective edges. Control plates had only fungal pathogens at one edge of the plate. Both control and test plates were incubated at 25 ± 2 °C for seven days. The diameter of radial growth of the fungal pathogens grown without and with antagonist fungus was determined, and the percentage growth inhibition was calculated as %growth inhibition = (A − B)/A × 100, where A represents the growth of the fungal pathogen in the control plate in cm, and B refers to the fungal growth in the test plate in cm [45].

## 3. Results

### 3.1. Morphological Characterization of Isolates

**Figure 1:**
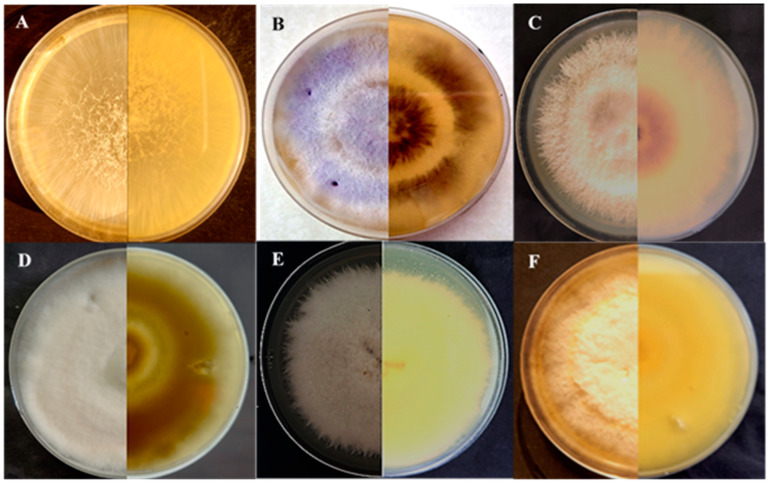
Front and back images of *Fusarium* cultures. (**A**) *F. fujikuroi*; (**B**) *F. annulatum*; (**C**) *F. oxysporum*; (**D**) *F. equiseti*; (**E**) *F. thapsinum*; and (**F**) *F. incarnatum*. Images were taken after 14 days of growth.

Ninety pure culture colonies with the apparent characteristics of *Fusarium* were isolated from all the samples. The surface characteristics, reverse color, colony color, and diameter of the fungal isolates are shown in Table 1 and ratings were determined based on previously published studies [22,36]. Surface and reverse characteristics are shown in Figure 1. Based on this morphological characterization, we determined that fungal isolates from this study consist of six different species: *F. equiseti*, *F. oxysporum*, *F. incarnatum*, *F. annulatum*, *F. fujikuroi* and *F. thapsinum. F. oxysporum* and *F. equiseti* were the predominant species in all the diseased samples.

**Table 1:** Morphological characteristics of different *Fusarium* species.

Isolate	Front Color	Surface Traits	Back Color	Colony Diameter after 7 Days	*Fusarium* spp.
AB 9	purple	cottony	purple	55 mm	*F. oxysporum*
AB 10	white	dense cottony	creamy white	55 mm	*F. equiseti*
AB 12	white	dense cottony	creamy white	45 mm	*F. thapsinum*
AB 13	white	dense cottony	golden brown	55.5 mm	*F. equiseti*
AB 25	white with a hint of pink	cottony	purple	55.2 mm	*F. oxysporum*
AB 26	purple and white	cottony	purple taupe	50 mm	*F. annulatum*
AB 27	white	cottony	ivory	45 mm	*F. fujikuroi*
AB 39	ivory	granular cottony	ivory	44 mm	*F. incarnatum*

**Figure 2:**
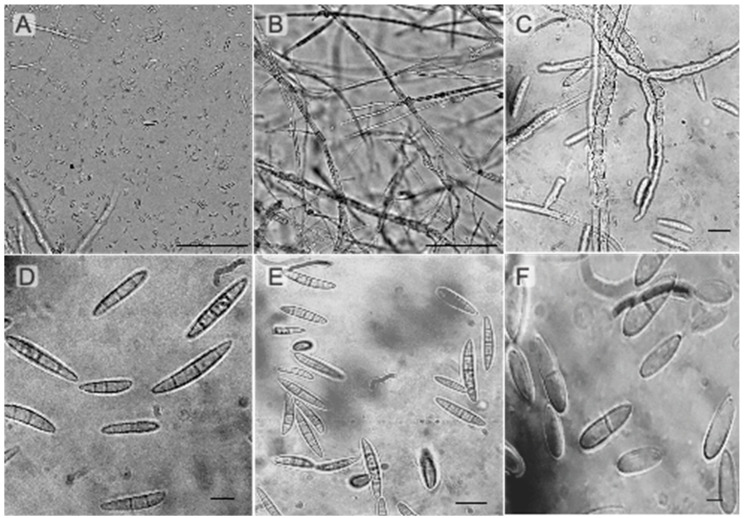
Representative photos of *Fusarium* mycelia and spores were taken using a binocular compound microscope (Bausch & Lomb Galen). (**A**) Macroconidia spores of *F. equiseti*; (**B**) mycelia of *F. equiseti*; (**C**) macroconidia spores of *F. equiseti*; (**D**) macroconidia spores of *F. oxysporum*; (**E**) macroconidia spores of *F. oxysporum*; (**F**) macroconidia spores of *F. incarnatum*. Scale bars: (**A**,**B**) = 100 µm; (**C**–**E**) = 25 µm; (**F**) = 10 µm.

*Fusarium* isolates categorized in the *F. fujikuroi* species complex (FFSC) produced moderately slender macroconidia with little curvature and did not produce *chlamydospores. F. fujikuroi* is placed in this category, producing club-shaped or copious ovate microconidia in shorter chains from monophialides and monophilalides with flattened bases comprising zero to one septum [46]. The *F. incarnatum* and *F. equiseti* species complex (FIESC) includes *Fusarium* isolates producing macroconidia with a crescent form or dorsiventral curvature. *F. incarnatum* placed in FIESC consisted of macroconidia with three to seven septa, slightly curved or straight, and fusiform with conical basal cells and a pointed apex [47,48]. Microscopic analyses of *F. equiseti* showed smooth branched and cylindrical hyphae, oval microconidia consisting of 0-1 septa, and curved and tapered macroconidia with 2-5 septa and elongated apical cells (Figure 2). *Fusarium* placed in the *F. oxysporum* species complex (FOSC) produced curved macroconidia, fusiform, with pointed, tapering, sometimes hooked apical cells and distinctly pedicellate basal cells consisting of three to five septa. Chlamydospores were also formed. *F. oxysporum* had branched, cylindrical, and smooth hyphae with ellipsoidal to cylindrical, straight, or curved microconidia bearing 0-1 septa. Macrocondia were pointed at both ends, consisting of 2-4 septa (Figure 2). *Fusarium oxysporum* and *F. equiseti* isolates were more prevalent among all the isolates from different locations taken from diseased leaves. Therefore, these two species were used for further experiments.

### 3.2. Molecular Characterization of Isolates

We confirmed morphologically identified isolates using *ITS* region sequence analysis. *ITS* sequences were compared to already published sequences of reference species. All the sequences showed 95–100% sequence identity with previously published sequences present within the NCBI GenBank database. *ITS* sequences from this work were used to assign species names according to references, submitted to NCBI GenBank, and accession numbers were obtained (Table 2). Our results indicated the presence of six species collected from different regions of Pakistan, including *F. thapsinum*, *F. annulatum*, *F. fujikuroi*, *F. incarnatum*, *F. oxysporum* and *F. equiseti*.

**Table 2:** *Fusarium* isolates used in this study and *ITS* sequence information.

Sample	Isolate	NCBI Percent Identity	*Fusarium* spp.	NCBI Accession	Isolation Site in Pakistan	Specimen Isolation
1	AB 9	99.75%	*F. oxysporum*	PP268016	all sampling regions	Leaves
2	AB 10	100%	*F. equiseti*	PP268017	all sampling regions	Leaves
3	AB 12	100%	*F. thapsinum*	PP268019	Sahiwal	Leaves
4	AB 13	100%	*F. equiseti*	PP268020	all sampling regions	Leaves
5	AB 25	100%	*F. oxysporum*	PP268021	all sampling regions	Leaves
6	AB 26	100%	*F. annulatum*	PP268022	Kamalia	Leaves
7	AB 27	100%	*F. fujikuroi*	PP268023	Chichawatni	Leaves
8	AB 39	100%	*F. incarnatum*	PP268018	Pakpattan	Leaves

### 3.3. Evolutionary Analysis by Maximum Likelihood Method for ITS and TEF 1α Sequences

**Figure 3:**
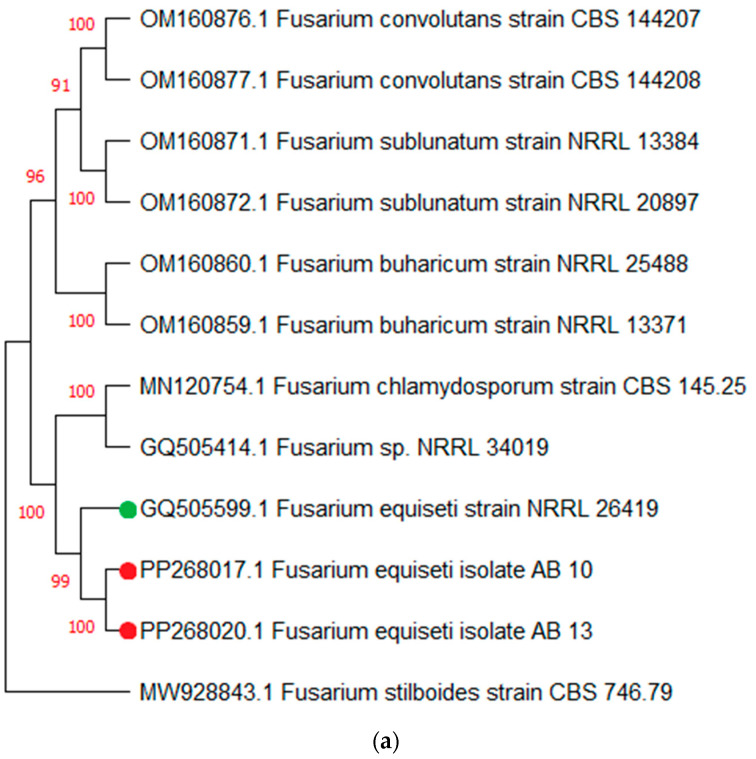

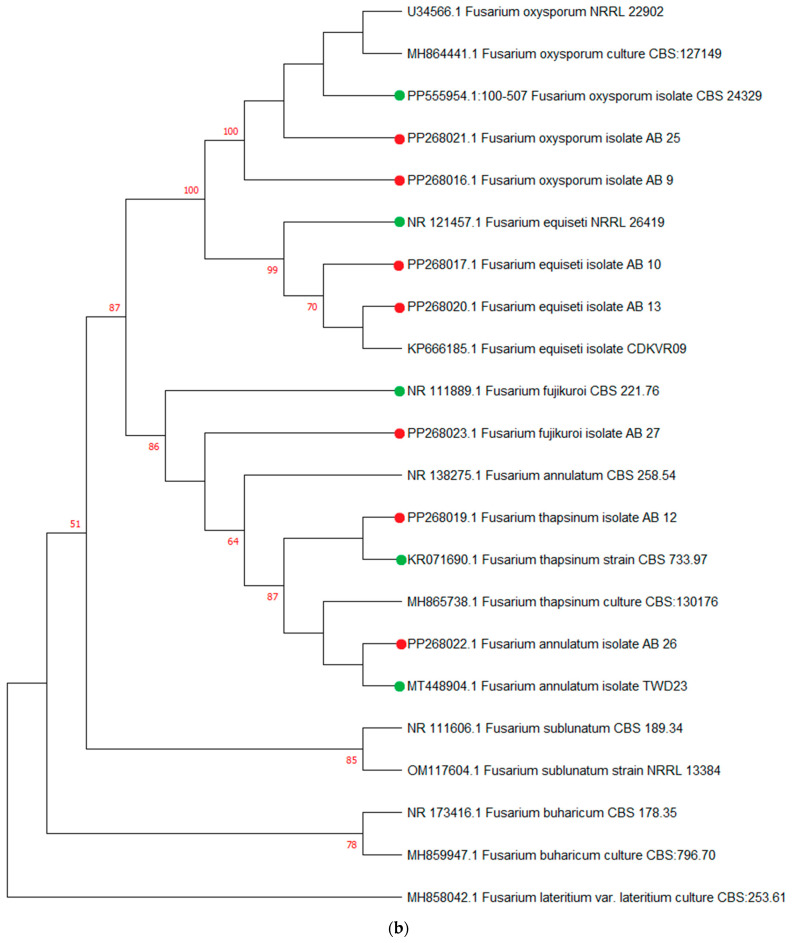
(**a**) Phylogenetic tree of partial *TEF* gene sequences for the evolutionary relationship of isolates by Maximum Likelihood method. Sequences shown in red are isolates from this study. *TEF* sequence from *Fusarium stilboides* was used as an outgroup. (**b**) Phylogenetic tree of partial *ITS* gene sequences for the evolutionary relationship of isolates by Maximum Likelihood method. Sequences shown in red are isolates from this study. GenBank accession numbers of *ITS* sequences are followed by the species name from which the sequences originated. *ITS* sequence from *Fusarium lateritium* was used as an outgroup. For both trees, red circles indicate isolation from Pakistan and green circles are database-derived sequences.

The evolutionary history was inferred using the Maximum Likelihood method and Jukes–Cantor model. The tree with the highest log likelihood (-2013.04) is shown (Figure 3). The percentage of trees in which the associated taxa clustered together is shown next to the branches. Initial tree(s) for the heuristic search were obtained automatically by applying Neighbor-Join and BioNJ algorithms to a matrix of pairwise distances estimated using the Jukes–Cantor model, and then selecting the topology with a superior log likelihood value. *Fusarium stilboides* was used as an outgroup for the *TEF-1α* gene phylogenetic tree. *Fusarium lateritium* was used as an outgroup for *ITS* sequence phylogenetic tree analysis and subsequent identification. Isolates from this study are marked red and type material from Genbank is marked green.

A phylogenetic tree of seven *ITS* sequences showed that isolates grouped with their respective *Fusarium* reference sequences (Figure 3). Isolates were also distributed in clades based on related species and showed evolutionary relationships among different species of *Fusarium*. The sequences of *Fusarium* isolates formed the same cluster with *F. equiseti* and *F. oxysporum* but in separate sub-clusters. There was a total of 460 positions in the final dataset. The results showed that isolates AB 9 and AB 25 nested with *F. oxysporum*, while isolates AB 10 and AB 13 nested with *F. equiseti*. Isolate AB 12 nested with *F. thapsinum*, isolate AB 26 with *F. annulatum*, isolate AB 27 with *F. fujikuroi*, and isolate AB 39 with *F. incarnatum*.

### 3.4. Fusarium Pathogenicity Test

**Figure 4:**
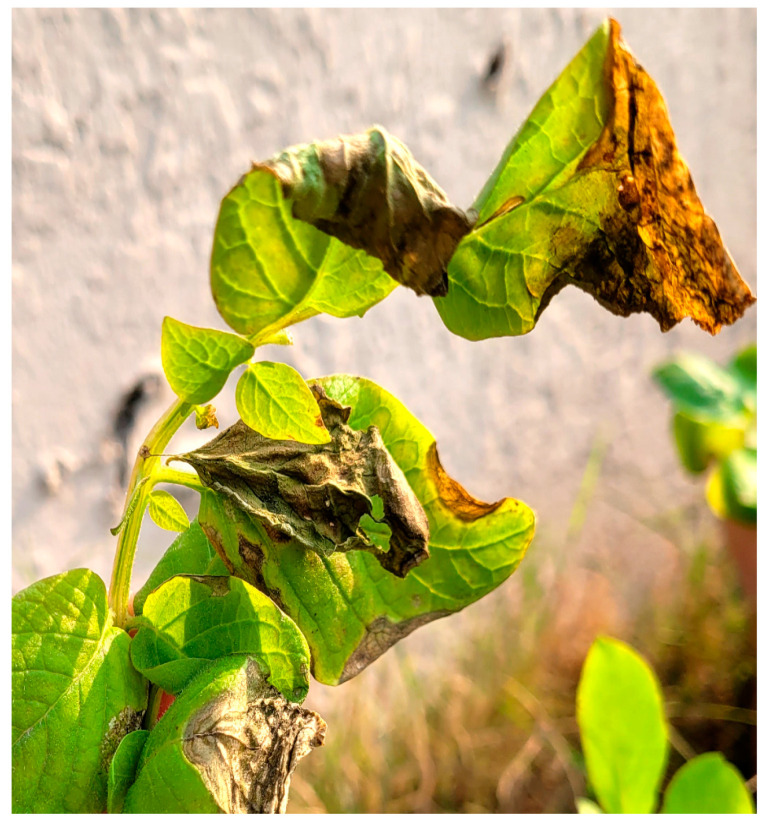
Symptoms of development of *F. oxysporum* on potato leaves. Photos were taken of greenhouse-grown plants 7 days after inoculation with *F. oxysporum*.

The pathogenicity of isolated fungi was confirmed using Koch’s postulates with greenhouse-grown Kuroda plants. Disease symptoms developed 7–14 days following inoculation and were visualized as light green to yellowish discoloration of leaves and necrosis (Figure 4), followed by wilting to death of the whole plant. When the collar region of the plant was cut vertically, the vascular bundles showed brownish discoloration. The potato plants were observed daily to follow disease progress. The most rapid development of symptoms started on day 7, caused by *F. oxysporum* isolates AB 9 and AB 25, followed by *F. equiseti* isolates AB 10 and AB 13, which induced symptoms 14 days after inoculation. Non-inoculated and water-inoculated control plants did not develop any symptoms. Plants inoculated with isolates of species *F. thapsinum*, *F. annulatum*, *F. fujikuroi*, and *F. incarnatum* did not show any wilting symptoms. The disease index was calculated using symptom scores 15 days post inoculation. Isolates of *F. oxysporum* showed wilting and yellowing of leaves with a disease index of 30 and 33.33 for isolates AB 9 and AB 25, respectively. *F. equiseti* isolates AB 10 and AB 13 developed symptoms on leaves to a lesser degree with disease indexes of 27.7 and 25, respectively. However, the complete foliage of the inoculated plants with both *F. oxysporum* and *F. equiseti* withered and all plants inoculated with these isolates were dead at the end of the trial. The pathogens were re-isolated from infected leaves and resembled the original culture in morphology.

Additional potato genotypes were tested with pathogenic *Fusarium* isolates, and based on physical observations, it was noted that AGB red and AGB CH2 exhibited only moderate symptoms compared to AGB pink and Kuroda, each of which had severe symptoms that led to plant death (Table 3). On this basis, AGB red and AGB CH2 were rated as resistant varieties compared to AGB pink and Kuroda.

**Figure 5:**
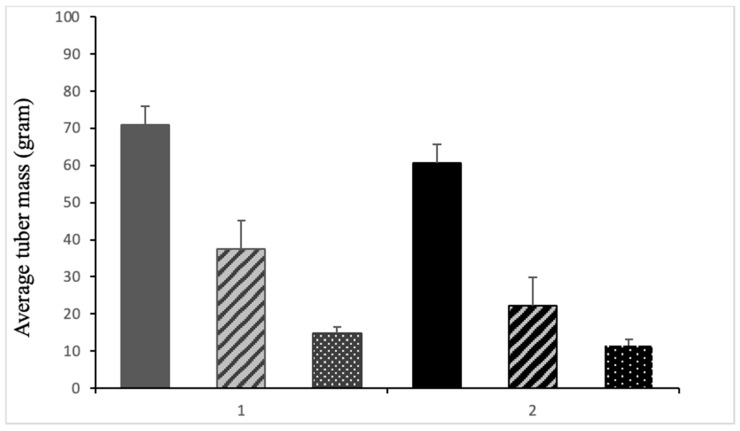
Series one represents the AGB red variety and series 2 represents the Kuroda variety. Solid bars in both series represent the control plants; the striped bar represents the average tuber mass in grams of plants treated with *Fusarium equiseti*; and the dotted bar represents the average tuber mass of plants treated with *Fusarium oxysporum*.

Potato cultivars AGB red and Kuroda were selected based on their resistance and susceptibility, respectively, against *F. equiseti* and *F. oxysporum*. Compared to control plants, plants treated with *F. equiseti* and *F. oxysporum* showed a reduction in tuber production, and their average mass was less than that of control plants. Among both *Fusarium* species, *F. oxysporum* resulted in lower tuber mass than *F. equiseti* (Figure 5).

### 3.5. Antagonistic Effect of Trichoderma on Fusarium Strains

**Figure 6:**
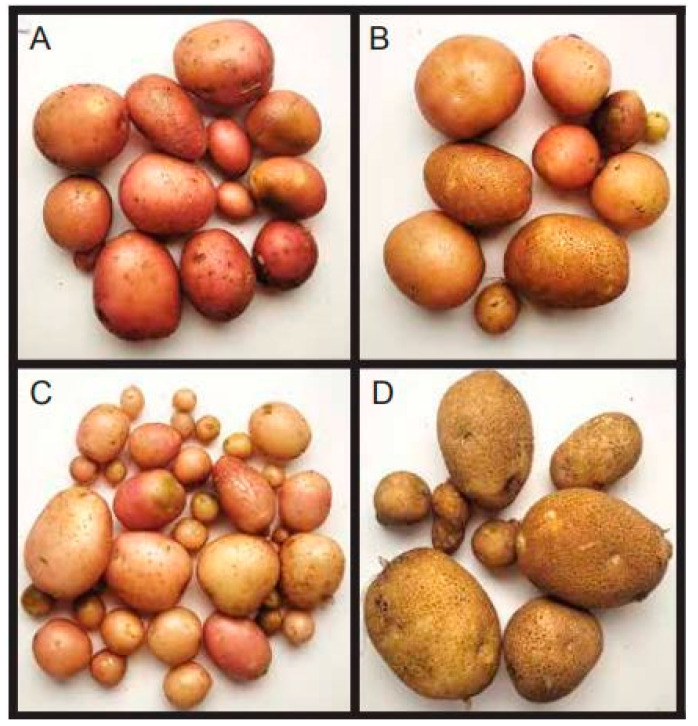
Tubers from greenhouse-grown plants inoculated with *Fusarium oxysporum*. (**A**) Kuroda; (**B**) AGB pink; (**C**) AGB red; (**D**) AGB CH2.

**Figure 7:**
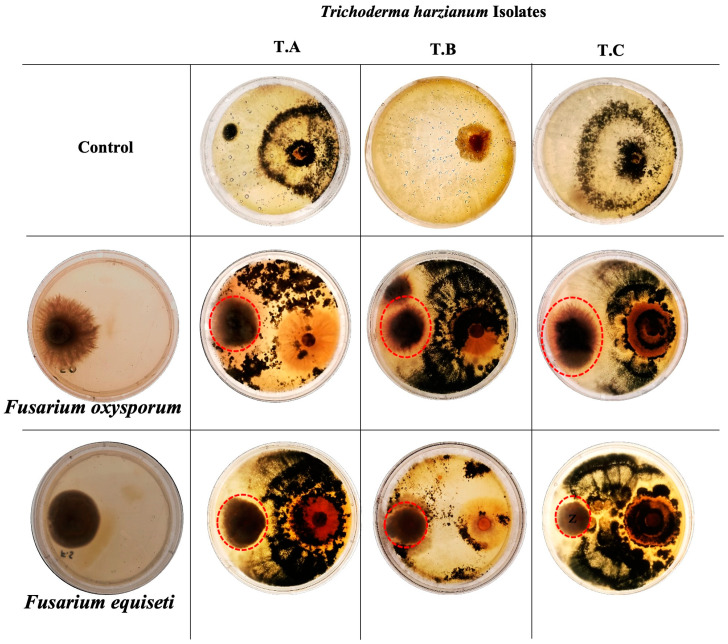
Inhibitory effect of *Trichoderma harzianum* on *Fusarium* growth (red circles). The upper row shows control for *T. harzianum* T.A, T.B and T.C and the left column shows control of *Fusarium oxysporum* and *equiseti*. The second row shows *Trichoderma harzianum* with *F. oxysporum*, while the lower panel shows *T. harzianum* with *F. equiseti*. Control shows the development of fungal pathogens in the absence of *T. harzianum* and vice versa.

To test the impact of the biocontrol fungus *Trichoderma harzianum* on the growth of collected *Fusarium* isolates, we grew both *T. harzianum* and *Fusarium* on plates and monitored their growth (dual-culture assay). We found that *T. harzianum* inhibited the growth of both *F. oxysporum* and *F. equiseti*, but to different degrees (Figure 6, Table 4). In the dual-culture assay, *T. harzianum* showed faster growth than pathogenic fungi. After two days of incubation, *T. harzianum* began restricting the growth of the pathogen as determined by visual observation (Figure 7). At five days, *T. harzianum* occupied most of the growth media. In the case of *F. oxysporum*, *T. harzianum* showed maximum inhibition of 65.21% and for *F. equiseti*, *T. harzianum* inhibited growth by 89.12%.

## 4. Discussion

*Fusarium*, a fungal species belonging to Ascomycota, is a prominent genus of over 1500 species. Several strains are reported for their pathogenicity to animals or plants [49]. Fusarium wilt is a soil-borne infection that ideally grows and infects plants at 34 °C or below 17 °C. Fusarium wilts reduce the yields of various economically important crops, such as fruits, cereals, and vegetables. Symptoms appear as marginal yellowing, sometimes chlorosis of adult plants, root rot, and damping off in young seedlings [50]. The pathogen causing wilting in potatoes collected throughout the major potato-growing region of Pakistan was isolated, characterized, and later confirmed as *F. oxysporum* and *F. equiseti* based on morphological descriptions and *ITS* and *TEF* sequencing. Six species of *Fusarium* were identified among isolates in this study. *Fusarium oxysporum* and *F. equiseti* were predominant in all the wilted potato leaf samples. According to previous studies, *F. oxysporum* and *F. equiseti* are the most common species isolated from potato wilt-diseased samples. Our results are in line with previous work that identified *F. oxysporum* as the dominant species with 50% isolation frequency, followed by *F. equiseti* at 20%, *F. redolens* at 20% and *F. acuminatum* with 10% isolation frequency [12]. However, we did not find *F. redolent* and *F. acuminatum* in our study. Instead, we found other *Fusarium* species including *F. incarnatum*, *F. fujikuroi*, *F. thapsinum*, and *F. annulatum*.

The present study evaluated *F. oxysporum* and *F. equiseti* isolates for their pathogenicity on multiple potato genotypes. We identified the role of *F. equiseti* for the first time in Pakistan along with *F. oxysporum* in the wilting of potatoes through re-isolation and pathogenicity assays following Koch’s postulates. Initial symptoms were first recorded for *F. oxysporum*, indicating it is more virulent than *F. equiseti*. *Fusarium oxysporum* was reported to cause wilting and devastating damage in Hidaka, Kochi, Japan, in 2008 [51]. Mousa et al. [52] reported *Fusarium oxysporum* as a causal agent for Fusarium wilt in sweet potato genotypes, and Cui et al. [53] reported *Fusarium equiseti* as a causal agent of wilting in potatoes in Zhengyi City, China. In Pakistan, most *Fusarium equiseti* species causing wilting in tomatoes have been reported from the northern area of Pakistan, Khyber-Pakhtunkhwa [54]. If we compare all the studies of wilt, *F. oxysporum* is the pathogen that is identified the most. However, we have determined that *F. equiseti* can also cause wilt in potatoes and can be found in high frequency in potato fields in Pakistan.

In addition to the clear identification of two wilt pathogens via Koch’s postulates, other *Fusarium* isolates were identified along with diseased samples. In the present study, *ITS* sequencing identified different isolates of *Fusarium*, including *F. incarnatum, F. thapsinum*, *F. fujikuroi*, and *F. annulatum*, along with *F. oxysporum* and *F. equiseti*.

Sequencing data showed different isolates of *Fusarium*, but not all of the *Fusarium* species were found to be pathogenic on potatoes. Our pathogenicity assay showed that *F. oxysporum* was more virulent than *F. equiseti*. The virulence assay on potato genotypes revealed that Kuroda was more susceptible than AGB pink, AGB CH2, and AGB red. AGB red was the most resistant among these four genotypes. Evaluation of tuber production revealed that *F. oxysporum* caused more reduction in mass, leading to an overall decrease in yield compared to *F. equiseti.*

During the last 3 to 4 decades, research has been reported on establishing biocontrol of Fusarium plant diseases [49]. Species of *Trichoderma* have been reported to be able to be used as a biocontrol agent against Fusarium wilt of many field crops, including potato, the efficiency of which may vary due to different agricultural and climatical conditions [55,56]. Laboratory and greenhouse experiments are vital for initial evaluation as preliminary tests [57]. In the work presented here, *Trichoderma harzianum* was evaluated for its antagonistic effect against *F. oxysporum* and *F. equiseti*. The results showed that *T. harzianum* can be a biocontrol agent against *F. oxysporum* and *F. equiseti*. The repressing effect of *Trichoderma harzianum* against *F. oxysporum* and *F. equiseti* may be due to antibiosis or competition.

## 5. Conclusions

This study is the first report of *F. equiseti* causing potato wilting in Punjab, Pakistan. Based on the phenotypical observation of plants during 2021 and 2022 in mostly potato-growing regions in the Punjab province of Pakistan, symptoms of Fusarium wilt were commonly found in the field compared to other fungal pathogens. Excessive use of appropriate fungicides might also be the reason for the excessive prevalence of disease incidence. Different isolates of *Fusarium* were identified through sequencing of the *ITS* and *TEF* regions; their pathogenicity was confirmed, and their effect on tuber production was evaluated. Among all the *Fusarium* species, our findings depict that *F. oxysporum* and *F. equiseti* are most common and that *F. oxysporum* is more virulent. Research on emerging diseases of crops has been carried out or is currently being performed in the developed world. However, there are relatively few research contributions from developing countries like Pakistan, where a changing climate is having the most dramatic effect. Exploring the emergence of new pathogens and pathogen genotypes in developing countries is essential so that scientists can track changes over time and provide recommendations for disease control appropriately.

## Figures and Tables

**Table 3:** Tuber production of greenhouse-grown plants inoculated with *Fusarium*.

Sample	Variety	Disease Severity	Number of Tubers	Tuber Skin Color
1	Kuroda	Severe	13	Red
2	AGB Red	Moderate	38	Red
3	AGB Pink	Severe	10	Red
4	AGB CH2	Moderate	8	White

**Table 4:** Inhibition of pathogen growth by antagonistic *Trichoderma harzianum*.

*T. harzianum*	Percentage Growth Inhibition ^1^	
	*Fusarium oxysporum*	*Fusarium equiseti*
A	54.96% ± 0.53	63.16% ± 0.71
B	65.21% ± 0.47	73.68% ± 0.58
C	55.27% ± 0.69	89.12% ± 0.83

^1^ Data represented in the table are mean values and standard deviation (*n* = 3).

## Data Availability

The isolates are available.
